# Editorial: Neuro-inspired sensing and computing: Novel materials, devices, and systems

**DOI:** 10.3389/fncom.2023.1126493

**Published:** 2023-01-12

**Authors:** Hongwei Tan, Zhong Sun, Xiaojian Zhu

**Affiliations:** ^1^Department of Applied Physics, Aalto University, Espoo, Finland; ^2^Institute for Artificial Intelligence, School of Integrated Circuits, Beijing Advanced Innovation Center for Integrated Circuits, Peking University, Beijing, China; ^3^Ningbo Institute of Materials Technology and Engineering, Chinese Academy of Sciences, Ningbo, China

**Keywords:** neuromorphic sensing and computing, neuroscience of pain, reservoir network, efficient spiking algorithm, arithmetic memristor

With the increasing amount of environmental and scientific data, energy-efficient and high-performance sensing, processing, and memory technologies for the tremendous data are essentially required (Mennel et al., 2020; Lanza et al., 2022). Learning from biological system is always the right way (Gu et al., 2020). Neuromorphic sensing and computing technologies have attracted enormous attention from academia and industry, due to their potential applications in intelligent sensing, parallel processing, and cognitive computing with low power consumption (Zhu et al., 2018; Sun et al., 2019; Wang et al., 2020; Liao et al., 2022). As the basis of human perception and cognition, the biological sensory neural networks with distributed and hierarchical topological structure, as well as parallel computing architecture have inspired researchers to design various artificial sensory-processing systems and algorithms toward perceptual and cognitive intelligence (Fazeli et al., 2019; Tan et al., 2021; Yu et al., 2022).

So far, breakthroughs in neuromorphic hardware (materials, devices, and systems, etc.) and software (machine learning algorithms, etc.), have facilitated the development of artificial sensory neural systems, making continuous progress toward cognitive sensing and computing. We believe that continued efforts in conceiving and exploring novel neuromorphic materials, devices, architectures, and algorithms are essential to achieving the ambitious goal of perceptual and cognitive intelligence.

This Research Topic “*Neuro-inspired sensing and computing: Novel materials, devices, and systems*” launched recently aims to highlight the most recent advances and achievements in neuro-inspired intelligent sensing and computing from researchers with multidisciplinary and interdisciplinary backgrounds. Below is the abstract information.

Neuroscience of pain: Neck pain is a worldwide health problem. Clarifying the etiology and providing effective interventions are challenging for the multifactorial nature of neck pain. As an essential component of cervical spine function, the sensorimotor control system has been extensively studied in both healthy and pathological conditions. Qu et al. from Nanchang University in China, Aalborg University in Denmark, and Northumbria University in UK provide a short review on neck pain from perspectives of proprioception, sensorimotor control system, neural plasticity, and potential interventions. This mini review may provide inspirations and new ideas to design artificial tactile and pain perception systems.

Reservoir network for image recognition: Liquid state machine (LSM) is a type of recurrent spiking network with a strong relationship to neurophysiology and has achieved great success in time series processing (Maass, 2011). However, the computational cost of simulations and complex dynamics with time dependency limit the size and functionality of LSMs. Dai et al. from Tohoku University in Japan present a large-scale bioinspired LSM with modular topology. They integrated the findings on the visual cortex that specifically designed input synapses can fit the activation of the real cortex and perform the Hough transform (Ballard, 1981), a feature extraction algorithm used in digital image processing, without additional cost. From their results, the proposed structure can not only significantly reduce the computational complexity but also achieve higher performance compared to the structure of previous reported networks of a similar size, as well as better robustness against system damage than the small-world and random structures. The proposed computationally efficient method may greatly contribute to future applications of reservoir computing.

Efficient competitive rate-based algorithm: Based on Competitive Spiking Neural Network (CSNN), Cachi et al. from Virginia Commonwealth University in US, Universidad de Córdoba in Spain, and Polish Academy of Science propose a rate-based algorithm equivalent to CSNN but that is much simple and well-suited to be run on regular computers. Based on the test results with MNIST and Fashion-MNIST, CRBA has reduced computational cost by up to three-orders of magnitude without reducing accuracy compared with CSNN. CRBA can be used for efficient deployment of CSNN on neuromorphic computers.

Arithmetic memristor: Implementation of arithmetic logic operations taking advantage of the non-linear characteristics of memristor can significantly improve the energy efficiency and simplify the complexity of peripheral circuits. Xin et al. from Northeast Normal University in China demonstrate an arithmetic logic unit function using a lateral volatile memristor based on layered 2D tungsten disulfide (WS2) materials and some combinational logic circuits. Mechanism and performance of the 2D memristive devices are comprehensively studied and characterized, showing a potential way to develop 2D memristive devices for future arithmetic logic applications.

These typical research works on the neuroscience understanding of pain, neural network model, algorithm, and memristive hardware are important for developing novel intelligent sensing and cognitive computing technologies. Learning from neuroscience, we believe more and more novel neuromorphic sensing and computing materials, devices, algorithms, and systems will be explored. We hope this Research Topic focusing on “*Neuro-inspired sensing and computing: Novel materials, devices, and systems*” will further initiate fruitful discussions and deep thinking in the community. We look forward to continuous progresses and breakthroughs in this emerging field of cognitive sensing and computing. In the end, we thank all the contributing authors for their novel research works and publications. We also thank the editorial team of “Frontiers in Computational Neuroscience” for their efforts in organizing this Research Topic.

## Author contributions

All authors listed have made a substantial, direct, and intellectual contribution to the work and approved it for publication.

## References

[B1] Ballard D. H. (1981). Generalizing the hough transform to detect arbitrary shapes. Pattern. Recog. 13, 111–122. 10.1016/0031-3203(81)90009-1

[B2] Fazeli N. Oller M. Wu J. Wu Z. Tenenbaum J. B. Rodriguez A. (2019). See, feel, act: hierarchical learning for complex manipulation skills with multisensory fusion. Sci. Robot. 4, eaav3123. 10.1126/scirobotics.aav3123 33137764

[B3] Gu L. Poddar S. Lin Y. Long Z. Zhang D. Zhang Q.. (2020). A biomimetic eye with a hemispherical perovskite nanowire array retina. Nature 581, 278–282. 10.1038/s41586-020-2285-x 32433619

[B4] Lanza M. Sebastian A. Lu W. D. Le Gallo M. Chang M.-F. Akinwande D.. (2022). Memristive technologies for data storage, computation, encryption, and radio-frequency communication. Science 376, eabj9979. 10.1126/science.abj9979 35653464

[B5] Liao F. Zhou Z. Kim B. J. Chen J. Wang J. Wan T.. (2022). Bioinspired in-sensor visual adaptation for accurate perception. Nat. Electron. 5, 84–91. 10.1038/s41928-022-00713-1

[B6] Maass W. (2011). “Computability in context,” in Liquid State Machines: Motivation, Theory, and Applications (World Scientific publishing), 275–296.

[B7] Mennel L. Symonowicz J. Wachter S. Polyushkin D. K. Molina-Mendoza A. J. Mueller T. (2020). Ultrafast machine vision with 2D material neural network image sensors. Nature 579, 62–66. 10.1038/s41586-020-2038-x 32132692

[B8] Sun Z. Pedretti G. Ambrosi E. Bricalli A. Wang W. Ielmini D. (2019). Solving matrix equations in one step with cross-point resistive arrays. Proc. Natl. Acad. Sci. U. S. A. 116, 4123–4128. 10.1073/pnas.1815682116 30782810 PMC6410822

[B9] Tan H. Zhou Y. Tao Q. Rosen J. van Dijken S. (2021). Bioinspired multisensory neural network with crossmodal integration and recognition. Nat. Commun. 12, 1120. 10.1038/s41467-021-21404-z 33602925 PMC7893014

[B10] Wang Z. Wu H. Burr G. W. Hwang C. S. Wang K. L. Xia Q.. (2020). Resistive switching materials for information processing. Nat. Rev. Mater. 5, 173–195. 10.1038/s41578-019-0159-3

[B11] Yu J. Wang Y. Qin S. Gao G. Xu C. Wang Z. L.. (2022). Bioinspired interactive neuromorphic devices. Mater. Today 60, 158–182. 10.1016/j.mattod.2022.09.012

[B12] Zhu X. Li D. Liang X. Lu W. D. (2018). Ionic modulation and ionic coupling effects in MoS2 devices for neuromorphic computing. Nat. Mater. 18, 141–148. 10.1038/s41563-018-0248-5 30559410

